# Immune Checkpoint Inhibitors in Lung Cancer: Role of Biomarkers and Combination Therapies

**DOI:** 10.7759/cureus.8095

**Published:** 2020-05-13

**Authors:** Tun Zan Maung, Huseyin Ekin Ergin, Mehwish Javed, Evelyn E Inga, Safeera Khan

**Affiliations:** 1 Internal Medicine, California Institute of Behavioral Neurosciences and Psychology, Fairfield, USA; 2 Medicine, California Institute of Behavioral Neurosciences and Psychology, Fairfield, USA; 3 Internal Medicine, LaSante Health Center, Brooklyn, USA

**Keywords:** lung cancer, immune checkpoint inhibitors

## Abstract

Lung cancer is the leading cause of cancer-related death worldwide, with a poor prognosis. Despite aggressive treatment, progression-free survival (PFS) and overall survival are limited. Recently, various kinds of immune checkpoint inhibitors (ICIs) have emerged for several cancers, targeting PD1, PDL1, and CTLA-4. ICIs have made a significant breakthrough in cancer and revolutionized the management of cancer including lung cancer. However, there are a lot of controversies regarding which group of patients is most suitable to be treated with ICIs in terms of monotherapy, combination, and predictive biomarkers. We reviewed various kinds of studies, such as meta-analysis, randomized control trials, multi-center cohort studies, and case-control studies from PubMed written in English from the last five years. ICIs have significant benefits in the overall survival compared with traditional chemotherapy. Patients with a higher level of PDL1 expression and high tumor mutational burden (TMB) have a higher response rate, and those with EGFR-/ALK- were better than those with EGFR+/ALK+. The patient who responded to immunotherapy completely can still maintain the efficacy after two years of treatment. Neoadjuvant immunotherapy in patients with resectable non-small cell lung cancer resulted in a 45% major pathology response (MPR) and 40% downstaging. Combined therapy (ICIs + chemotherapy) was better than chemotherapy alone, irrespective of PD‐L1 expression. A combination of ICIs such as CTLA‐4 and PD‐1/PD‐L1 improved PFS as well. Radiochemotherapy ahead of ICIs is promising as well. However, ICIs combined with EGFR/ALK‐TKI (tyrosine kinase inhibitor) are not suggested for the time being. PDL1 expression, TMB, and EGFR/ALK mutations are promising predictive biomarkers. Gut microbiota, galectin-3, and intensity of CD8 cell infiltration are other potential predictive biomarkers. These are very important in the future management of lung cancers as they can prevent unnecessary toxicities and cost of treatment.

## Introduction and background

Lung cancer is at the top of the list for cancer-related death worldwide [1]. It is a tumor with a poor prognosis. Non-small cell lung cancer (NSCLC) is approximately 80% of all lung cancer cases, and the majority of these cases were diagnosed at an advanced stage [2]. Despite the aggressive treatment of early and locally advanced disease, SCLC often relapses. First-line chemotherapy provides reasonable response rates in advanced disease, but progression-free survival (PFS) and overall survival (OS) are limited. New drugs such as some targeted therapies and immune therapies are promising in SCLC. Some molecular targeting agents such as epidermal growth factor receptor (EGFR), tyrosine kinase inhibitors (TKIs), and anaplastic lymphoma kinase (ALK) inhibitors have a good response in patients with EGFR or ALK mutations [3,4]. However, most patients with NSCLC do not have these oncogenic drivers, and treatment options are limited to cytotoxic chemotherapy for these patients. Recently, various kinds of immune checkpoint inhibitors (ICIs) have been established for several cancers, targeting PD1, PDL1, and CTLA-4 [5-7]. ICIs have made a significant breakthrough in cancer and revolutionized the management of cancer. Currently, clinical evidence supporting the efficacy of checkpoint blockade in NSCLC has been very significant. Pembrolizumab, nivolumab, and atezolizumab have promising results in lung cancer and are approved for treating lung cancer. Some other agents are still in trial. There is enough evidence from recent trials that these improve disease-free survival (DFS) and OS in lung cancer. Pembrolizumab was already approved as the first-line agent in lung cancer with PDL1 expression of more than 50%. But, pembrolizumab was found effective in only less than half of the patients with a PDL1 expression of more than 50% [8,9]. Checkpoint inhibitors have become first-line therapy for most of the patients with metastatic disease, but there are a lot of controversies regarding ICIs [10]. Which patient group is most beneficial from this kind of treatment, such as histology types, PD1, or PDL1 expression? Is it worth checking predictive biomarkers that indicate a good response? Do combination therapies such as ICIs and chemotherapy, ICIs and TKIs, ICIs and radiotherapy, and a combination of ICIs bring better outcomes? Should ICIs be rechallenged in relapse cases? In this traditional review, we are going to look into the impact of PD1, PDL1 expression, predictive biomarkers, and combination therapy on DFS and OS of lung cancer.

## Review

Methods

We used PubMed to collect data for this review. We included various kinds of studies such as meta-analysis, randomized control trials, multi-center cohort studies, and case-control studies. We used keywords as “lung neoplasm” and “immunotherapy in combination” to search papers published in the last five years. A total of 50 research papers that were in English were extracted, and 29 papers were shortlisted after both abstracts and full-text screening [10-38].

Inclusion and Exclusion Criteria

Papers on the effects of ICIs on lung cancer in terms of DFS, OS, predictive biologic markers, and combination therapies were used. Various studies such as meta-analysis, randomized control trial, multi-center cohort studies, and case-control studies published in English in the last five years were included. Studies published in other languages and published before the previous five years were excluded.

Results

We extracted 50 articles by using keywords (“lung neoplasm” and “immunotherapy in combination”) from the PubMed database. Among the 50 articles, we looked specifically into DFS, OS, predictive biologic markers, and combination therapies of 29 papers and reviewed both abstract and full text. All studies were in English. 

Discussion

Mechanisms of Immune Checkpoint Inhibitors

ICIs are a breakthrough in various kinds of cancer including lung cancer. Here, we would like to elaborate on the mechanism of checkpoint inhibitors. The immune system generally recognizes and destroys cancer cells. If immunity becomes low, tumor cells adapt to the immune system and escape destruction, which results in uncontrolled tumor cell growth. The human immune system involves innate and adaptive components. Natural killer cells are essential components of the innate immune system. In an adaptive immune system, T lymphocytes (CD4 and CD8) have an important role. Tumor cell uses many complex ways to escape from recognition and destruction from the immune system. To escape from the immune system, tumor cells mainly use T-cell inhibitory pathways such as cytotoxic T-lymphocyte antigen 4 (CTLA-4), programmed death 1 (PD1), lymphocyte antigen gene 3 (LAG-3), and suppression of NK cell activities. ICIs counteract the mechanisms used by tumor cells to suppress the immune system targeting PD1, PDL1, and CTLA-4. Currently, two PD-1 (pembrolizumab, nivolumab), one PDL1 (atezolizumab) inhibitor, and one ipilimumab (CTLA-4) inhibitor are approved [39].

**Figure 1:**
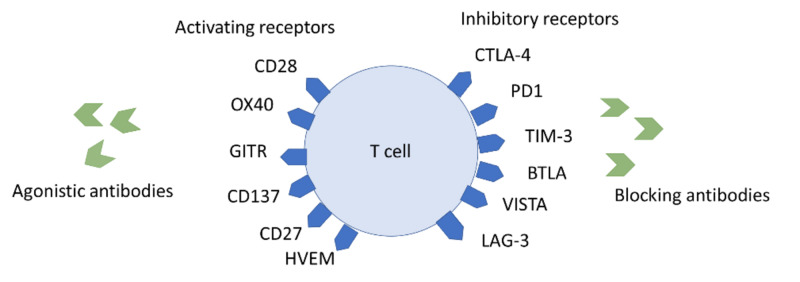
Mechanism of immune suppression and immune checkpoint inhibitors

Figure 1 shows the mechanism of immune suppression and ICIs.

Overall Survival and Progression-Free Survival of Immune Checkpoint Inhibitors

ICIs have significant benefits in OS compared with traditional chemotherapy in the case-control analysis [13]. There was no considerable difference in OS and DFS between PD1 and PDL1 inhibitors, but odd ratios of pembrolizumab (PDL1 expression ≥ 50% ) and nivolumab (PDL1 expression ≥ 1%) were higher than that of atezolizumab [12]. Patients with a higher level of PDL1 expression and high tumor mutational burden (TMB) have a higher response rate. Checkmate 02610 showed that nivolumab could not be used as a first‐line treatment even if their PD‐L1 expression level were more than 50%. In the ATLANTIC34 study with durvalumab (third‐line or more advanced NSCLC), ORR, PFS, and OS were better in a patient with high expression of PD‐L1 than those with a low expression of PD‐L1, and those with EGFR-/ALK- were better than those with EGFR+/ALK+. The patients who responded to immunotherapy completely maintained the efficacy after two years of treatment. CTLA‐4 inhibitor ipilimumab alone showed no significant effect on lung cancer [21]. Neoadjuvant immunotherapy in patients with resectable NSCLC resulted in a 45% major pathology response (MPR) and 40% downstaging. MPR is found in both PDL1-positive and PDL1-negative tumors but more in patients with a higher TMB. It is more effective in early staging. [10,14]. Table 1 shows the studies discussing the survival of ICIs.

**Table 1:** Studies focusing on the survival of ICIs ICI, immune checkpoint inhibitor; MPR, major pathology response; OS, overall survival

N0.	Author	Year of publication	Purpose of the study	Intervention studied	Result/conclusion
1.	Broderick and Bott [10]	2019	Effects of neoadjuvant ICIs	ICIs	Improve MPR and OS
2.	Almutairi et al. [12]	2019	Efficacy/safety of PD1 and PDL1 inhibitors	ICIs	Pembrolizumab and nivolumab rank top in OS
3.	Faehling et al. [13]	2019	The response of ICIs to a tumor mass	ICIs	Increase response in lower tumor stage and small tumor mass
4.	Lanuti et al. [14]	2019	Major pathological response, toxicities	ICIs	Improved major pathological response

Combination of Immune Checkpoint Inhibitors

Combination immunotherapy improves OS and PFS compared with single-agent therapy. The KEYNOTE-021 study including patients with advanced NSCLC treated aggressively before reported that pembrolizumab plus ipilimumab showed evidence of antitumor activity with significant toxicity [17]. In the KEYNOTE-18949 and KEYNOTE-407 studies, pembrolizumab + chemotherapy vs. chemotherapy was tested for non-squamous and squamous cell carcinoma, and the outcome showed that combined therapy was better than chemotherapy alone irrespective of PD‐L1 expression. The IMPower131 trial also proved that with first‐line atezolizumab combined with chemotherapy in advanced squamous NSCLC, long‐term survival was better than chemotherapy alone. The Checkmate012 study showed the same results for nivolumab plus chemotherapy. Ipilimumab plus chemotherapy showed improved PFS, but the benefits are limited and the toxicity is obvious [21]. A combination of etoposide and cisplatin or of carboplatin and atezolizumab has become standard for small cell lung cancer [27]. In the PACIFIC study with durvalumab consolidation therapy after radical chemoradiation therapy, PFS was significantly longer than the placebo group. In the KEYNOTE-001 and LUN 14‐179 studies, patients with advanced NSCLC previously treated with radiotherapy showed longer PFS and OS with pembrolizumab treatment than those who had no radiotherapy before. Immunotherapy, combined with EGFR/ALK‐TKI, is not suggested as nivolumab combined with crizotinib in the Checkmate37056 study and durvalumab combined with osimertinib in the TATTON study encountered severe treatment-related adverse events. When it comes to immunotherapy plus antivascular therapy, regardless of EGFR/ALK sensitive mutations, or liver metastasis, atezolizumab + carboplatin + paclitaxel + bevacizumab significantly prolonged the PFS of patients. In terms of a combination of ICIs such as CTLA‐4 and PD‐1/PD‐L1, the Checkmate22724 study showed that a PFS rate with nivolumab + ipilimumab is better than chemotherapy in patients with TMB ≥ 10, but with no benefit in patients with TMB ≤ 10 [21]. Combination of PD-1 and CTLA-4 blockade and agents targeting IDO1, B7-H3, VEGF, and NGFR show promising results [38]. Table 2 shows some of the selected studies on ICIs.

Predictive Biomarkers

Immunotherapy is very effective in lung cancers, but not all patients with lung cancer benefit from it. The response rates of these ICIs were from 14% to 20% in unselected patients. It is, therefore, essential to find predictive biomarkers for the selection of patients who are likely to benefit from the ICI [30]. Further studies are needed on this. In patients with an ultra-high PDL1 expression, pembrolizumab response lasts longer, and there is a less chance of treatment failure in the late phase. PDL1 expression levels might be a predictive biomarker for immunotherapy benefit [11,31]. ICIs have a good response in lung cancers with high TMB. First-line ICIs have a limited effect in EGFR+/ALK+ NSCLC [16]. Male, ever-smoker, and positive PDL1 are indicators of benefit to ICIs in metastatic NSCLC [20]. PDL1 expression and TMB are potential predicted biomarkers [25,26,37]. In patients with negative or low/intermediate expression of galectin-3 in tumor cells, pembrolizumab has an early and durable response. Therefore, galectin-3 is an interesting predictive marker and can be useful in a better patient selection for immunotherapy. A large multicenter trial is ongoing to confirm this [29]. EGFR mutations are associated with low response rates in anti‐PD‐1/PD‐L1 treatments, and different microbiota and microbiomes, and different biochemical and metabolic profiles may affect the efficacy of ICI treatments as well [30]. Many predictive biomarkers are found to be associated with treatment responses to immune checkpoint blockade therapies, including tumor mutational load, DNA mismatch repair deficiency, the composition of the gut microbiome, the intensity of CD8+ cell infiltration, and intratumoral PDL1 expression. Patients with high TMB had a better response, prolonged clinical benefit, and PFS when treated with ICIs. In one randomized phase III trial with stage IV or recurrent NSCLC, nivolumab as the first-line therapy was found not superior to chemotherapy in PFS among patients whose tumor had a PDL1 expression of ≥5%. However, nivolumab had a higher response rate and longer PFS than chemotherapy among patients with high TMB overall. Lung cancer has high TMB when compared with other cancers [33]. The integration of molecular markers and immune-PET (positron emission tomography) imaging may be a potentially effective strategy for patient selection [35]. Some of the studies about predictive biomarkers are shown in Table 3.

**Table 2:** Studies discussing predictive biomarkers ICI, immune checkpoint inhibitor; TMB, tumor mutational burden; EGFR, epidermal growth factor receptor; NSCLC, non-small cell lung cancer; TKI, tyrosine kinase inhibitor; ALK, anaplastic lymphoma kinase; PARPi, poly(ADP-ribose) polymerase inhibitors

No.	Author	Year of publication	Purpose of the study	Intervention Study	Result/conclusion
1.	Edahiro et al. [11]	2019	PDL1 expression	ICIs	Risk of treatment failure decreases if high PDL1 expression
2.	Wojas-Krawczyk et al. [15]	2019	Predictive biomarkers	ICIs	PDL1, TMB, and microbiome are effective biomarkers
3.	Proto et al. [16]	2019	Predictive biomarkers	ICIs	If PDL1 is negative and there is decrease in TMB, little benefit from ICIs, in EGFR + NSCLC, ICIs has limited activity, ICI + TKI in EFGR/ALK-positive raised concern, in EGFR and ALK + NSCLC, atezolizumab + platinum - base chemotherapy + bevacizumab is a potential treatment
4.	Bylicki et al. [18]	2019	Predictive biomarkers	ICIs	PDL1 and TMB are promising biomarkers
5.	El-Osta and Jafri [20]	2019	Predictive biomarkers	ICIs	Male, smoker, and PDL1 + benefit from ICIs
6.	Hendriks et al. [23]	2019	Predictive biomarkers	ICIs	DLL3-antibodies or combinations of PARPi and immunotherapy could be very promising
7.	Wang et al. [25]	2019	Predictive biomarkers	ICIs	TMB and PDL1 are promising biomarkers
8.	Duma et al. [26]	2019	Predictive biomarkers	ICIs	PDL1 is a promising biomarker
9.	Capalbo et al. [29]	2019	Predictive biomarkers	ICIs	Low glectin-3 tumor expression make a durable response to ICIs
10.	Xia et al. [30]	2019	Predictive biomarkers	ICIs	EGFR + a low response to ICIs, microbiota affect efficacy of ICIs
11.	Camidge et al. [31]	2019	Predictive biomarkers	ICIs	PDL1 expression and increase TMB increase ICIs response
12.	Pu et al. [33]	2018	Predictive biomarkers	ICIs	TMB, DNA mismatch repair deficiency, gut microbiome, the intensity of CD8+ cell infiltration, PDL1 are potential biomarkers, increased response of ICIs in increased TMB, lung cancer has increased TMB, abnormal gut microbiome due to use of antibiotics before ICIs decrease the effect, no enhanced effect of ICIs in EGFR + group, pembrolizumab increase OS and PFS in EGFR–/ALK– lung cancer
13.	Marmarelis and Aggarwal [38]	2018	Predictive biomarkers	ICIs	TIGIT, LAG-3, and cellular therapy are future biomarkers

Limitations

There were a few limitations to this review paper. Only papers that were written in English were reviewed; therefore, there is a chance of missing some vital information published in any other language. We were able to retrieve only a few randomized controlled trials that proved to be a limitation for an in-depth analysis of the topic. The full text was not available for all of the papers; therefore, details about the research methodology of those articles were not known, and quality check of all papers was not performed.

## Conclusions

ICIs are very promising in the management of cancer patients, including both small cell lung cancer and NSCLC. It prolongs both OS and PFS. Furthermore, a combination of ICI and chemotherapy is more effective, and some ICIs become part of the first-line treatment. It was also proved that combination of ICIs targeting different receptors more prolong OS and PFS. ICIs combined with radiotherapy are promising as well. With TKIs, only a few of ICIs are beneficial, and some combinations cause more toxicities and have no benefits. However, it is also noted that all lung cancer patients are not responsive to ICIs, and predictive biomarkers have an essential role here for the selection of patients who may get the most benefits. PDL1 expression, TMB, and EGFR/ALK mutations have promising results but still need more evidence. Gut microbiota, galectin-3, and intensity of CD8 cell infiltration are other potential predictive biomarkers. These are very important in the future management of lung cancers as they can prevent unnecessary toxicities and cost of treatment. This paper will definitely help doctors who are dealing with lung cancer cases. In the future, more clinical trials will be needed to look into the aforementioned issues, and we believe that in the future, immunotherapy will make lung cancers to be a chronic disease rather than a deadly disease.
